# High Variability in Implementation of Selective-Prevention Services for Cardiometabolic Diseases in Five European Primary Care Settings

**DOI:** 10.3390/ijerph17239080

**Published:** 2020-12-04

**Authors:** Christos Lionis, Marilena Anastasaki, Antonios Bertsias, Agapi Angelaki, Axel C. Carlsson, Hrafnhildur Gudjonsdottir, Per Wändell, Anders Larrabee Sonderlund, Trine Thilsing, Jens Søndergaard, Bohumil Seifert, Norbert Kral, Niek J De Wit, Monika Hollander, Joke Korevaar, François Schellevis

**Affiliations:** 1 Clinic of Social and Family Medicine, School of Medicine, University of Crete, 70013 Heraklion, Greece; anastasakimarilena@yahoo.gr (M.A.); antonisbertsias@yahoo.gr (A.B.); a.angelaki@uoc.gr (A.A.); 2 Division of Family Medicine and Primary Care, Department of Neurobiology, Care Sciences and Society, Karolinska Institutet, 14183 Huddinge, Sweden; axel.carlsson@ki.se (A.C.C.); per.wandell@ki.se (P.W.); 3 Academic Primary Health Care Centre, Stockholm Region, 11365 Stockholm, Sweden; 4 Centre for Epidemiology and Community Medicine, Region Stockholm, 11365 Stockholm, Sweden; hrafnhildur.gudjonsdottir@sll.se; 5 Research Unit of General Practice, Department of Public Health, University of Southern Denmark, 5000 Odense C, Denmark; asonderlund@health.sdu.dk (A.L.S.); tthilsing@health.sdu.dk (T.T.); jsoendergaard@health.sdu.dk (J.S.); 6 Institute of General Practice, First Faculty of Medicine, Charles University, 128 00 Prague 2, Czech Republic; seifert@terminal.cz (B.S.); norbert.kral@seznam.cz (N.K.); 7 Julius Center for Health Sciences and Primary Care, University Medical Center Utrecht, 3584 CX Utrecht, The Netherlands; n.j.dewit@umcutrecht.nl (N.J.D.W.); m.hollander-2@umcutrecht.nl (M.H.); 8 Nivel Netherlands Institute for Health Services Research, 3513 CR Utrecht, The Netherlands; j.korevaar@nivel.nl (J.K.); f.schellevis@nivel.nl (F.S.); 9 Department of General Practice, Amsterdam Public Health Research Institute, Amsterdam University Medical Centers, location VUmc, 1081 HV Amsterdam, The Netherlands

**Keywords:** cardiometabolic diseases, cardio-vascular diseases, feasibility study, prevention, primary care, risk reduction

## Abstract

(1) Background: Cardiometabolic diseases are the most common cause of death worldwide. As part of a collaborative European study, this paper aims to explore the implementation of primary care selective-prevention services in five European countries. We assessed the implementation process of the selective-prevention services, participants’ cardiometabolic profile and risk and participants’ evaluation of the services, in terms of feasibility and impact in promoting a healthy lifestyle. (2) Methods: Eligible participants were primary care patients, 40–65 years of age, without any diagnosis of cardiometabolic disease. Two hundred patients were invited to participate per country. The extent to which participants adopted and completed the implementation of selective-prevention services was recorded. Patient demographics, lifestyle-related cardiometabolic risk factors and opinions on the implementation’s feasibility were also collected. (3) Results: Acceptance rates varied from 19.5% (n = 39/200) in Sweden to 100% (n = 200/200) in the Czech Republic. Risk assessment completion rates ranged from 65.4% (n = 70/107) in Greece to 100% (n = 39/39) in Sweden. On a ten-point scale, the median (25–75% quartile) of participant-reported implementation feasibility ranged from 7.4 (6.9–7.8) in Greece to 9.2 (8.2–9.9) in Sweden. Willingness to change lifestyle exceeded 80% in all countries. (4) Conclusions: A substantial variation in the implementation of selective-prevention receptiveness and patient risk profile was observed among countries. Our findings suggest that the design and implementation of behavior change cardiometabolic programmes in each country should be informed by the local context and provide some background evidence towards this direction, which can be even more relevant during the current pandemic period.

## 1. Introduction

Cardiometabolic disease (CMD), including cardio-vascular disease (CVD) and type-II diabetes mellitus (T2DM), represents the most common cause of death worldwide, accounting for more than 17.3 million deaths globally every year [1,2]. CMD has also been included among the clinical entities that predict serious consequences of COVID-19 during the current pandemic. Although statistics indicate a significant decline of CVD morbidity and mortality in most European countries, issues relevant to quality of life and health care expenditure associated with CVD remain considerably cumbersome [1]. Nonetheless, current evidence indicates that up to 80% of CMDs can be prevented or delayed through lifestyle changes, mainly including those related to diet, exercise, smoking and alcohol consumption [3,4,5,6].

The United States Institute of Medicine classifies preventive strategies in four categories: indicated prevention; care-related prevention; universal prevention; and selective prevention [7]. Selective prevention aims to identify high-risk, asymptomatic individuals in the general population and offer them preventative strategies. Evidence from modeling studies and systematic reviews suggests that the selective prevention of CMD can help reduce the burden of disease in the general population [8,9]. In terms of CMD-preventative efforts, primary care and general practitioners (GPs) are in the front lines, with the European Society of Cardiology (ESC) guidelines stating that “GPs have a unique role in identifying individuals at risk of, but without established CVD, and assessing their eligibility for intervention” [6]. In the past ten years, numerous evidence-based clinical guidelines have been developed and implemented in various settings around the world, with extensive CMD screening being particularly successful in the UK, New Zealand and Australia. However, it is not certain to what extent and with what effect this has been applied in European primary care settings.

The present study is part of this European SPIM-EU project (http://spimeu.org/). SPIM-EU seeks to contribute to the reduction of CMD morbidity and mortality in Europe by testing the feasibility of evidence-based implementation of selective-prevention services, and providing comprehensive tools for their implementation in primary care [10]. Within the framework of SPIM-EU, an expert consensus meeting was conducted, and a set of statements representing the key characteristics of selective CMD prevention was proposed in order to develop a universal concept of selective CMD prevention that can guide implementation within European primary care [11]. The overall aim of the present study was to assess the feasibility of implementing CMD selective-prevention services in different European primary care systems (the Czech Republic, Denmark, Greece, the Netherlands and Sweden) and report on what we achieved, with a special focus on patient participation and acceptance rates. We also sought to report on participants’ CMD profile, their CVD-risk and their perception and barriers towards lifestyle modification.

## 2. Materials and Methods

### 2.1. Design and Settings

This was a descriptive study reporting on the feasibility of selective-prevention strategies in the participating countries.

The general design and critical determinants of the selective prevention services were informed by a consensus meeting with an international panel of 14 experts, who explored the relevant evidence from systematic literature reviews and surveys conducted within SPIM-EU [11]. The selective-prevention services basically included a CVD-risk assessment of primary care patients. In all countries, primary care practices were purposefully selected (with the exception of the Netherlands) to act as study sites. Specifically, ten practices participated in the Czech Republic (average practice size: 1900 persons), two in Denmark (1600 persons), three in Greece (1500 persons), five in the Netherlands (2350 persons) and one in Sweden (20,000 listed persons).

### 2.2. Participants

Participants were patients of the participating primary care practices in the five countries. In the Netherlands and Denmark, participants were randomly selected, while in the other settings a consecutive sampling was followed. The choice between the random or consecutive sampling of patients was based on the local availability of comprehensive patient listings, which could act as sampling frames. Patients were eligible to participate if they were between 40–65 years of age and had not been diagnosed with a CMD, such as hypertension, CVD, T2DM, chronic renal disease and/or hypercholesterolemia. Since this was a descriptive study assessing implementation feasibility, the patient-level sample size to estimate the rate of completed CVD-risk assessment was based on the rule of thumb of n = 30 or greater. As such, a convenience sample of 200 participants per country was set as a recruitment goal.

### 2.3. Procedures and Outcomes

Details regarding procedural aspects in each participating country are presented in Supplementary Table S1. The overall implementation of selective-prevention services consisted of patient invitation and CVD-risk assessment, and was delivered in a similar manner in all countries. It was up to each trial site, however, to determine and implement the most locally relevant patient identification and invitation methods, as well as CVD-risk assessment instrument. In particular, this implementation included the following:

1. **Identification and invitation of 200 participants**: Eligible participants were identified via primary care practice patient lists. They were invited to the study either personally by the staff of the practice (the Czech Republic, Greece, the Netherlands and Sweden), or—in the case of Denmark—via patients’ digital mailbox (a digital mail-system provided by the government for secure and direct communication between individuals, public authorities and other trusted organizations). The recruitment period ran from April through October 2018 at all sites.
2. **Initial assessment of participant CMD-risk profile**: An online questionnaire, developed and based on the European Social Survey (http://www.europeansocialsurvey.org), was used. The questionnaire was either administered to participants by practice personnel or research assistants during a face-to-face consultation (the Czech Republic, Greece, the Netherlands and Sweden) or completed by participants online (Denmark). The questionnaire recorded demographic characteristics (age, gender, education, work status, insurance and income) and lifestyle-related CMD-risk factors (smoking, alcohol consumption, physical activity and nutrition).
3. **Comprehensive CVD-risk assessment**: Each country selected a tool for CVD-risk measurement, based on the ESC or national guidelines [6]. Locally validated tools used in clinical practice in each country were selected to facilitate the local adaptation of the planned implementation. In the Czech Republic and Greece, country-adjusted versions of the European Heart SCORE were used [12]. In Sweden, Svenska Score (or SCORE Sweden) was selected [13]. In Denmark and the Netherlands, the modified Heartscore BMI score [14] and the Dutch Prevention Consultation Cardiometabolic Risk (PC CMR) [15] were used, respectively. Upon assessment, participants were verbally informed about their CVD-risk, and where relevant, provided with practical advice on how to reduce it.
4. **Participant evaluation of the implementation selective-preventive services**: On a ten-point Likert scale, participants were asked to assess the relevance, usefulness and feasibility of the selective-prevention services, as well as the extent to which it encouraged a healthier lifestyle. Participants’ willingness to change risk behavior, as well as any encountered barriers to lifestyle modification, were also assessed.

The study assessed the following parameters to evaluate the process and outcomes of this implementation in each setting:

1. Numbers and proportions of patients who accepted the invitation and completed their CMD-risk profiling (feasibility)
2. Numbers and proportions of participants who completed the comprehensive CVD-risk assessment (feasibility)
3. Participant-reported intervention relevance, usefulness, feasibility and impact in pursuing a healthier lifestyle, along with respective barriers (evaluation)

### 2.4. Statistical Analysis

Outcomes were summarized using descriptive statistics. Gender differences in terms of participants’ willingness to change lifestyle and barriers towards changing lifestyle were explored using X^2^ tests. The level of statistical significance was set at a = 0.05. Analysis was performed using SPSS (Version 23.0. Armonk, NY, USA: IBM Corp.).

### 2.5. Ethics

The study was approved by local bioethics committees in each country (Czech Republic: Ethics Committee of the General University Hospital, Prague 1946/16 S-IV; Greece: Research and Development Committee of the 7th Health Region of Crete 13685/09-08-17; Denmark: University of Southern Denmark’s list of approved studies journal nr. 18/5728; Sweden: Etikprövningsnämnden I Stockholm 2017/2053-31; and the Netherlands: University Medical Center Utrecht 17-702/C). All participants signed an informed consent form prior to their participation in the study.

## 3. Results

### 3.1. Recruitment

**Figure 1:**
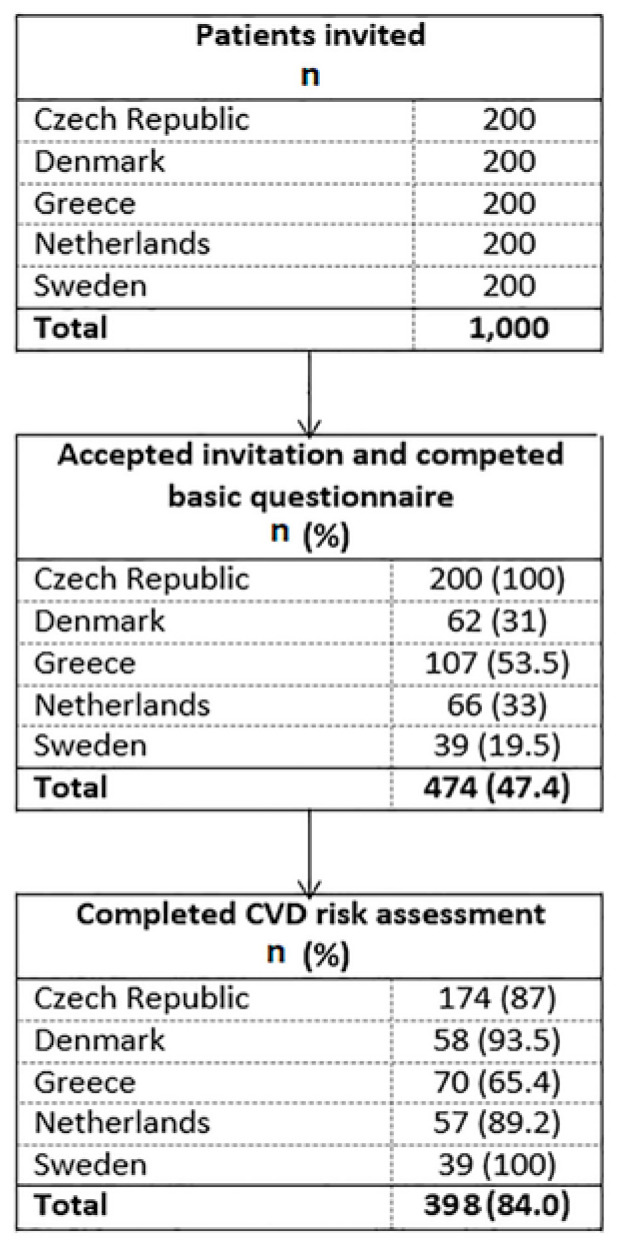
Study flow chart in the five European primary care settings.

Figure 1 shows the study flowchart. As per the recruitment goal, 200 eligible individuals were identified and invited to participate in each country (total N = 1000). Overall, less than half of invited individuals (47.4%, n = 474) accepted the invitation. Acceptance rates ranged from 19.5% (n = 39/200) in Sweden to 100% (n =200/200) in the Czech Republic. Sweden had the highest risk-assessment completion rate (100%, n = 39/39) among all countries, while Greece had the lowest (65.4%, n = 70/107). Across all sites, there was an 84.0% (n = 398/474) risk-assessment completion rate.

### 3.2. Participants

Demographic characteristics of participants who accepted the invitation (n = 474) are summarized in Table 1. Women were over-represented in all countries [Czech Republic: 60.5% (n = 121); Greece: 59.8% (n = 34); the Netherlands: 54.5% (n = 36); and Sweden: 69.2% (n = 27)] apart from Denmark (46.8%, n = 29). The mean age of participants ranged from 50 (± 8.8) years in the Czech Republic to 55.5 (± 6.3) years in Denmark.

In all countries except for Greece, most participants had completed university education [Czech Republic: 82.3% (n = 63); Denmark: 83.4% (n = 45); the Netherlands: 80.3% (n = 53); and Sweden: 100% (n = 39)]. In Greece, the majority of participants had completed secondary education (48.6%, n = 52). Most participants were working full-time, and were covered by health insurance. In the Czech Republic and Sweden, most participants reported an income above the country average [42.2% (n = 84) and 61.5% (n = 24), respectively]. In the Netherlands, most participants had an income equal to the national average (53.8%, n = 35), while in Greece the reported income was lower than the average (62.6%, n = 67).

Demographic characteristics of individuals who completed the CVD-risk assessment (n = 398) are presented separately in Supplementary Table S2, along with an analysis of gender and age differences between individuals who completed versus those who did not complete the CVD-risk assessment per country.

### 3.3. CMD-Risk Profiling

CMD-risk factors related to participant lifestyle are presented in Table 2 (n = 474). The prevalence of daily smoking ranged from 43% (n = 46) in Greece to 3.1% (n = 2) in the Netherlands. Long-term ex-smokers (quit over six months ago) accounted for 34.4% (n = 22) in the Netherlands, 33.3% in Denmark (n = 20), 20.5% in Sweden (n = 8), 17% (n = 34) in the Czech Republic and 15% in Greece (n = 16). Rates of never-smokers ranged from 32.7% (n = 35) in Greece to 71.8% (n = 28) in Sweden.

The median (IQR) number of standard alcoholic beverages consumed weekly was [7 (9)] in Greece, followed by Denmark [4 (8)], Sweden [3 (5)], the Netherlands [2 (7)] and the Czech Republic [2 (6)]. Additionally, more than 10% of participants in all countries stated that they drink four (for women) or five (for men) standard drinks on a single occasion at least once per week.

Rates of sedentary lifestyle ranged from 8.1% (n = 5) in Denmark to 19.6% (n = 21) in Greece. Roughly one out of four participants were classified as under-active (light or moderate exercise, not weekly) in the Czech Republic (31.6%, n = 62), Denmark (25%, n = 12), the Netherlands (20%, n = 13) and Sweden (25.6%, n = 10), while the rate was almost double that in Greece (48.1%, n = 51).

Daily vegetable consumption was reported by most participants in Sweden (82%, n = 32), the Netherlands (80%, n = 52) and Denmark (61.3%, n = 38), but not in the Czech Republic (44.2%, n = 88) or Greece (12.1%, n = 13). In all countries, daily fruit consumption was reported by half or more of participants, apart from Greece where the rate was 21.5% (n = 23). In the Czech Republic, Denmark and Greece, most participants reported fish consumption a few times per month [62.1% (n = 123), 50% (n = 31) and 67.3% (n = 72), respectively].

### 3.4. CVD-Risk Assessment

Table 3 presents CVD-risk scores for participants who completed the risk assessment tool used in each country (n = 398). The median (25–75% quartiles) of the country-adjusted European Heart SCORE was 1 (0–3) in Greece and 1 (0–2) in the Czech Republic. The median of the Svenska score in Sweden was, remarkably, zero [0 (0–1)]. In Greece, 11.4% (n = 8) of participants were found with increased CVD-risk (SCORE ≥ 5%). Respective rates for the Czech Republic and Sweden were 6.9% (n = 21) and zero. In Greece and the Czech Republic, 4.3% (n = 3) and 2.3% (n = 4) of participants respectively were classified in the highest CVD-risk category (SOCRE ≥ 10%). In Denmark, the median (25–75%) of the modified Heartscore BMI score was 2 (1–3), with 8.6% (n = 5) of participants classified as high CVD-risk. In the Netherlands, the median (25–75%) PC CMR score was 22 (13.5–39.5), with 21 (36.8%) participants found to be at high risk.

### 3.5. Participant Evaluation of the Intervention

**Figure 2:**
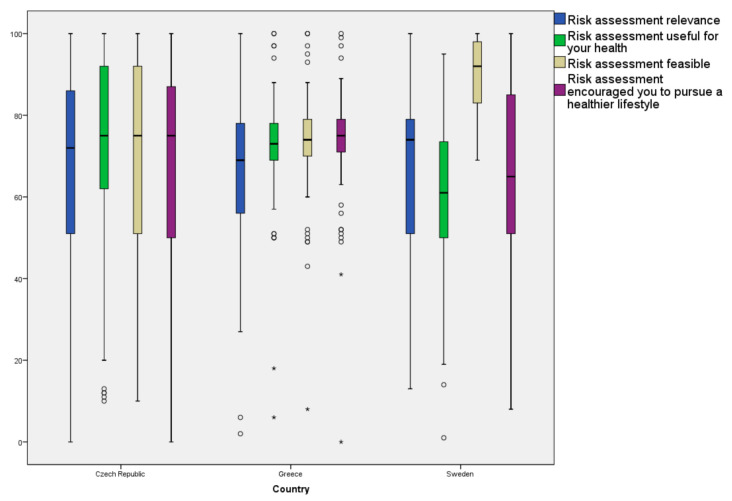
Participants’ evaluation of the risk assessment intervention. (Participants from Denmark and the Netherlands did not answer evaluation questions, despite their invitation to do so.)

Figure 2 shows participants’ evaluation of the selective prevention process assessed on a ten-point scale. In Sweden, intervention feasibility was assessed with the highest scores [median (25–75% quartile): 9.2 (8.2–9.9)], while usefulness received the lowest scores [median (25–75%): 6.1 (5–7.4)]. Greek participants assessed intervention’s ability to encourage a healthier lifestyle with the highest scores [7.6 (7.1–7.9)], and its relevance the lowest [5.9 (5.5–7.7)]. In the Czech Republic, all process evaluation dimensions received similar assessments, with median scores (25–75%) ranging from 7.2 (5.1–8.6) for relevance to 7.5 (6.2–9.2) for usefulness. Participants in Denmark and the Netherlands did not evaluate the risk assessment intervention, despite their invitation to do so.

### 3.6. Perception and Barriers Towards Lifestyle Modification

**Figure 3:**
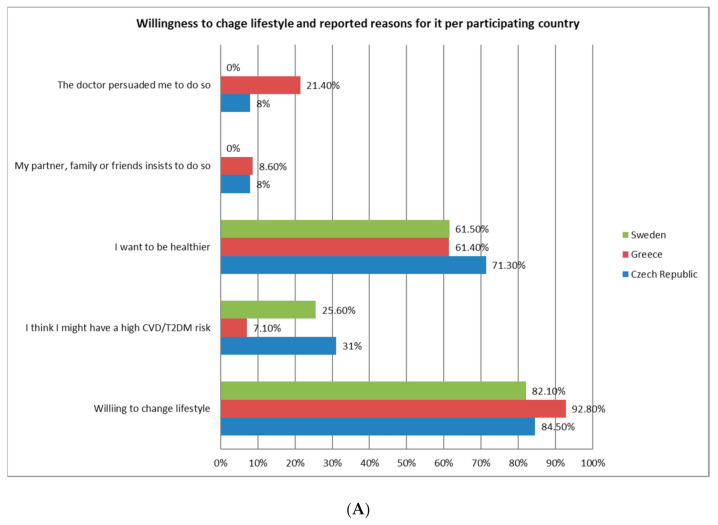

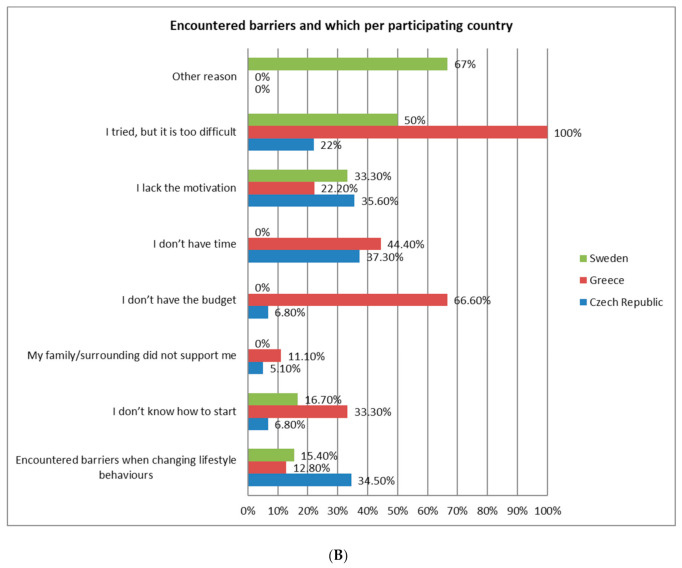
Willingness (**A**) and barriers (**B**) towards changing lifestyle among participants accepting risk assessment: (Despite invitation, participants from Denmark and the Netherlands did not answer evaluation questions). Abbreviations: CVD: cardiovascular disease, T2DM: type-II diabetes mellitus.

In response to the risk assessment, the vast majority of participants in the Czech Republic (84.5%, n = 147), Greece (92.8%, n = 64) and Sweden (82.1%, n = 32) reported that they were willing to change their lifestyle in order to reduce their CVD-risk (Figure 3A). The main motivation behind this willingness was related to their desire for better health [Czech Republic: 71.3% (n = 124); Greece: 61.4% (n = 43); and Sweden: 61.5% (n = 24)]. A secondary reason for the behavioral change specific to the Czech Republic was to lower an increased CVD-risk (31%, n = 51), while in Greece it was a doctor who motivated patients to change their lifestyle (21.4%, n = 15). In Sweden, lowering an increased CVD-risk was the only secondary reason for behavioral change (61.5%, n = 24). Participants in Denmark and the Netherlands did not respond to the above questions.

In the Czech Republic, 14 (8%) responded that they were willing to change lifestyle because their partner, family or friends convinced them [4 (3.6%) of these being females and 10 (15.9%) being males, *p* = 0.04]. No other statistically significant differences in terms of willingness to change lifestyle between males and females were observed in this country. In Greece, the proportion of females that replied that they were willing to change lifestyle because in order to be healthier (n = 31, 70.5%) was higher compared to males (n = 12, 46.2%; *p* = 0.044). No other significant differences were observed between males and females in terms of willingness to change in this country. In Sweden, no significant gender differences in the willingness to change lifestyle were noted.

About one out of three Czech participants (34.5%, n = 59) stated that they had encountered barriers in their attempt to change their lifestyle (Figure 3B). In Greece and Sweden, 12.8% (n = 9) and 15.4% (n = 6) reported similar barriers to lifestyle change, respectively. Lack of time (37.3%, n = 22) and motivation (35.6%, n = 21) were identified as central obstacles for the 59 Czech participants who had encountered barriers in their lifestyle-change attempts. In Greece, all nine (100%) participants stated that they had tried, but found it too difficult to start a healthier lifestyle. Other barriers reported by Greek participants included a lack of budget (66.6%, n = 6), time (44.4%, n = 4) and knowledge about where to start (33.3%, n = 3). In Sweden, four out of six (66.7%) participants who reported barriers to lifestyle change gave other reasons for not changing their lifestyle, followed by having tried but finding it too difficult (50%, n = 3), as well as a lack of motivation (33.3%, n = 2). Regarding the reported barriers to change, no significant differences were observed between males and females (*p* > 0.05 for all).

## 4. Discussion

### 4.1. Summary of Findings and Comparison with Literature

Our study indicated substantial cross-country variations in the implementation of selective CMD prevention services, as well as in participant receptiveness. Although our findings cannot be generalized within or between countries, these variations could be interpreted in terms of the differences in primary care systems included in our study, and as such, underline the necessity for European health policies and CMD prevention strategies, taking into account the particularities of local contexts.

Another important finding concerns the substantial differences between study participants regarding various lifestyle factors. Participants from Greece, followed by the Czech Republic, showed the most unfavorable health profile, which was also reflected in their CVD-risk scores. This finding is in agreement with international statistics, and warrants further attention [16]. Remarkably, no individuals with an elevated CVD risk were identified in Sweden, a country where the acceptance rate for participation was quite low. The favorable profile in Sweden may be partially attributed to the selective participation of respondents, who are often healthier than the average person. However, although Sweden has no primary prevention program, another possible explanation could be that the country is generally quick to adopt and incorporate healthy lifestyle guidelines. Indeed, this is further suggested by the fact that it is the first country with a daily smoking prevalence of below 10% [17].

Between-country variation was also observed in terms of participants’ acceptance of the selective-prevention services, with the Czech Republic and Greece being most open to intervention. This seems to be a positive finding, since participants in both countries presented an unfavorable health profile in terms of CVD risk. Although these variations may be partially attributed to differences in the invitation processes implemented in each country, it may also reflect (and perhaps specifically for Greece) the general lack of systematic preventive activities provided by interdisciplinary teams in primary care [18]. This finding may thus serve as a key message for health policy actions, especially in settings where coordinated primary care reforms are evolving.

The implemented selective-prevention services succeeded, providing 65% to 100% of participants with CVD-risk assessments, resulting in the identification of substantial proportions of high-CVD-risk individuals (6.9% to 36.8%). A similar Dutch study identified 64% of participants as being high-risk, with 22% of these classified as newly diagnosed patients suffering from various conditions such as hypertension, hypercholesterolemia and diabetes [19]. Furthermore, our intervention was generally perceived as useful and feasible by participants, with the vast majority indicating that, in response to the implementation, they would be willing to try a lifestyle modification program for CMD-risk reduction (82.1% to 92.8%).

Finally, the most commonly reported reason to adopt a healthier lifestyle was a desire to be healthy (61.4% to 71.3%). This finding is consistent with a recent systematic review conducted in the context of SPIM-EU, where prioritizing and feeling responsible for one’s own health were recognized as facilitators for participating in a CMD health check in primary care [20]. We are aware that willingness to be healthy is not a sufficient determinant for behavior change, and the literature is rich with interventions where several theoretical frameworks have been used in successful interventions. However, the most important lesson of this collaborative study concerns the high variations between countries regarding the implementation of CMD screening and its implications for health policy, education and research.

### 4.2. Strengths and Limitations

The main strength of this study is that it was designed to include and assess a set of characteristics and recommendations for a selective CMD-prevention program, formed on the basis of an expert consensus. However, the study had several limitations. Firstly, it was a descriptive study, using the rule of thumb to determine sample size, without formal statistical power calculations. As such, our study may have under-reported the true proportions of patients with increased CVD-risk, and our results certainly cannot be generalized within or between countries. However, the aim of the study was to report on the feasibility of implementing selective prevention, rather than to identify the magnitude of CVD in primary care. Our design does not also allow for any type of comparisons or causality determination. Moreover, the purposive selection of study sites (in four out of five settings) and differences in the patient sampling method chosen per country do not allow for a random representation of either practices or patients. Although the overall intervention followed the same principles of implementation, different ways of invitation, risk assessment tools and communication strategies were used, according to the diverse local contexts and the objectives of this study. Although this affects the comparability of participation and acceptance rates, as well as participants’ assessment of the intervention, it was intended to explore the variability of the selective prevention strategies in each setting. In any case, the assessment of the proportion of patients who completed the CVD-risk assessment in this study will facilitate the design and implementation of a future full-scale trial in each setting.

### 4.3. Study Implications

Our study revealed a substantial variation in the implementation, acceptance and completion of the evaluated selective-prevention services across the participating countries. Similar patterns have been reported in literature, with participation in health checks in primary care varying widely according to intervention type (e.g., response rates ranging from a low 1.2% for an online risk estimation to a high of 84% for T2DM screening) [19,20]. The country-specific and locally validated risk assessment tools will allow better adaptation of the intervention in local contexts. Barriers for CMD selective-prevention in primary care, including structural, organizational and attitudinal factors, may have a significant impact on patient participation [21], and should be taken into account before the design and implementation of full-scale interventions.

The action plan of the World Health Organization for the prevention and control of non-communicable diseases lists CMD-risk assessment and management among its five focus areas for priority interventions. Moreover, it acknowledges that further development of primary care services, together with public health services, is essential for improving health promotion, disease prevention, early detection and integrated care [2]. The issue of an evidence-based integration of public health and primary health care has also been discussed in local reports from Greece [22]. Within this study, a directed targeted implementation of a selective CMD prevention intervention in diverse primary care settings was conducted. Lessons learned can be used to refine similar interventions, accounting for specific factors that may influence implementation.

This study is particularly pertinent in Europe, where strategies for CVD prevention vary across countries and systems. In countries like Greece, major health care reforms are currently evolving in an effort to bring primary care to the forefront of the national health care system [23]. In other settings like the Czech Republic, nationwide selective prevention programs screening for CMD-risk in the general population are already available [24]. In countries such as the Netherlands, specific guidelines for selective CMD-prevention have been developed by the Dutch College of GPs to target the segment of the general population that is between 45 and 70 years of age and without any known risk factors [25]. In Denmark, national strategies for selective CMD prevention are lacking. By contrast, the Vasterbotten Intervention Program in Sweden has been integrated with primary care since 1985, with the population being invited to participate in a systematic risk-factor screening at the ages of 40, 50 and 60 [26]. Ultimately, and as part of SPIM-EU, this study has contributed to the formation of an evidence-based toolbox for the design and implementation of selective primary care initiatives targeting CMD. In a recently published article, 12 general recommendations are illustrated on how to best design and implement CMD selective-prevention [27].

## 5. Conclusions

This study demonstrated country-specific variations in the implementation and receptiveness of selective prevention interventions in five European countries where CMD is a major public health issue. Although our results are not directly comparable across study sites due to contextual and procedural differences, they still contribute to the existing evidence regarding the necessity of CMD selective prevention actions within each study country. Our findings also emphasize the need for European CMD prevention policies, tailored to local needs and contexts.

## Figures and Tables

**Table 1:** Demographic characteristics of individuals accepting the invitation per country.

Variable	Czech Republic (N = 200)	Denmark (N = 62)	Greece (N = 107)	Netherlands (N = 66)	Sweden (N = 39)
**Gender,** *n (%)*					
*Female*	121 (60.5)	29 (46.8)	34 (59.8)	36 (54.5)	27(69.2)
*Male*	79 (39.5)	33 (53.2)	43 (40.2)	30 (45.5)	12 (30.8)
**Age (years),** *mean (SD)*	50.0 (8.8)	55.5 (6.3)	52.7 (8.5)	54.0 (10.3)	51.1 (6.3)
**Education,** *n (%)*					
*None*	0 (0)	0 (0)	5 (4.7)	0 (0)	0 (0)
*Primary*	1 (0.5)	1 (1.9)	19 (17.8)	1 (1.5)	0 (0)
*Secondary*	34 (17.2)	8 (14.8)	52 (48.6)	12 (18.2)	0 (0)
*College/University*	163 (82.3)	45 (83.4)	31 (29)	53 (80.3)	39 (100)
**Work status,** *n (%)*					
*Full* *-time*	131 (65.5)	37 (59.7)	62 (57.9)	32 (48.5)	36 (92.3)
*Part* *-time*	30 (15)	11 (17.7)	22 (20.6)	17 (25.8)	2 (5.1)
*Pensioner*	11 (5.5)	8 (12.9)	9 (8.4)	12 (18.2)	1 (2.6)
*Unemployed*	4 (2)	4 (6.5)	14 (13.1)	3 (4.5)	0 (0)
*Disabled*	24 (12)	2 (3.2)	0 (0)	2 (3)	0 (0)
**Health insurance,** *n (%)*					
*Yes*	192 (96)	26 (41.9)	84 (79.2)	66 (100)	27 (69.2)
*No*	3 (1.5)	4 (6.5)	21 (19.8)	0 (0)	7 (17.9)
*Not applicable*	5 (2.5)	32 (51.6)	1 (0.9)	0 (0)	5 (12.8)
**Income compared to country’s average,** *n (%)*					
*Lower*	44 (22)	21 (34.4)	67 (62.6)	5 (7.7)	3 (7.7)
*Corresponding*	61 (30.7)	22 (36.1)	23 (21.5)	35 (53.8)	8 (20.5)
*Higher*	84 (42.2)	17 (27.9)	0 (0)	24 (36.9)	24 (61.5)
*Don’t know*	10 (5)	1 (1.6)	17 (15.9)	1 (1.5)	4 (10.3)

**Table 2:** Lifestyle-related cardiometabolic risk factors of individuals accepting invitation per country (n = 474).

Variable	Czech Republic (n = 200)	Denmark (n = 62)	Greece (n = 107)	Netherlands (n = 66)	Sweden (n = 39)
**Smoking,** *n (%)*					
*Never*	122 (61)	24 (40)	35 (32.7)	36 (56.3)	28 (71.8)
*Quit over 6 months* *ago*	34 (17)	20 (33.3)	16 (15)	22 (34.4)	8 (20.5)
*Quit less than 6 months* *ago*	3 (1.5)	1 (1.7)	1 (0.9)	1 (1.6)	1 (2.6%)
*Occasionally*	13 (6.5)	5 (8.3)	9 (8.4)	3 (4.7)	0 (0)
*Everyday*	28 (14)	10 (16.7)	46 (43)	2 (3.1)	2 (5.1)
**Drinks/week,** *median (min, max; IQR)*	2 (0, 40; 6)	4 (0, 60; 8)	7 (0, 46; 9)	2 (0, 70; 7)	3 (0, 30; 5)
**Consumption of ≥4 (female) or 5 (male) drinks on a single occasion,** *n (%)*					
*Everyday*	4 (2)	4 (6.5)	3 (2.8)	2 (3.1)	0 (0)
*Once a week*	29 (14.5)	8 (12.9%)	12 (11.3)	8 (12.5)	4 (10.3)
*Once a month*	48 (24)	18 (29)	11 (10.4)	8 (12.5)	8 (20.5)
*Rarely*	86 (43)	29 (46.8)	31 (29.2)	25 (39.1)	24 (61.5)
*Never*	33 (16)	3 (4.8)	49 (46.2)	21 (32.8)	3 (7.7)
**Physical activity,** *n (%)*					
*Sedentary (rarely/never)*	29 (14.7)	5 (8.1)	21 (19.6)	8 (12.1)	4 (10.3)
*Underactive (light/moderate* *, not weekly)*	62 (31.6)	12 (25)	51 (48.1)	13 (20)	10 (25.6)
*Regular-light (light, weekly)*	146 (74.1)	38 (67.9)	75 (70.1)	39 (60.9)	33 (84.6)
*Regular-moderate (moderate, weekly, ≤30 min/day)*	91 (46.4)	29 (52.7)	36 (33.6)	29 (44.6)	22 (56.4)
*Regular-vigorous (vigorous, weekly, ≤20 min/day)*	59 (30.1)	12 (23.5)	10 (9.3)	16 (24.2)	16 (41)
*Active-moderate (30 min moderate for ≥5 days/week)*	58 (29.4)	26 (47.3)	13 (12.1)	28 (42.4)	15 (38.5)
*Active-vigorous (20 min vigorous for ≥3 days/week)*	33 (16.8)	11 (20)	10 (9.3)	11 (17.5)	11 (28.2)
**Vegetable consumption,** *n (%)*					
*≤once/week*	22 (11.1)	2 (3.2)	32 (29.9)	0 (0)	2 (5.1)
*A few times/week*	89 (44.7)	22 (35.5)	62 (57.9)	13 (20)	5 (12.8)
*Once/day*	71 (35.7)	25 (40.3)	12 (11.2)	46 (70.8)	24 (61.5)
*≥twice/day*	17 (8.5)	13 (21)	1 (0.9)	6 (9.2)	8 (20.5)
**Fruit consumption,** *n (%)*					
*≤once/week*	21 (10.5)	9 (14.5)	27 (25.2)	2 (3.1)	5 (12.8)
*A few times/week*	71 (35.5)	24 (38.7)	57 (53.3)	17 (26.2)	12 (30.8)
*Once/day*	83 (41.5)	22 (35.5)	19 (17.8)	30 (46.2)	15 (38.5)
*≥twice/day*	25 (12.5)	7 (11.3)	4 (3.7)	16 (24.6)	7 (17.9)
**Fish consumption,** *n (%)*					
*A few times/month*	123 (62.1)	31 (50)	72 (67.3)	17 (26.2)	6 (15.4)
*Once/week*	56 (28.3)	19 (30.6)	26 (24.3)	33 (50.8)	14 (35.9)
*Twice/week*	13 (6.6)	9 (14.5)	9 (8.4)	12 (18.5)	13 (33.3)
*≥three times/week*	6 (3)	3 (4.8)	0 (0)	3 (4.6)	6 (15.4)
**Pastry consumption,** *n (%)*					
*≤once/week*	28 (14)	16 (25.8)	21 (20.2)	18 (27.7)	19 (48.7)
*A few times/week*	59 (29.5)	25 (40.3)	32 (30.8)	27 (40.9)	15 (38.5)
*Nearly every day*	41 (20.5)	15 (24.2)	20 (19.2)	9 (13.8)	5 (12.8)
*Everyday*	72 (36)	6 (9.7)	31 (29.8)	11 (16.9)	0 (0)

**Table 3:** Cardio-vascular risk scores among individuals accepting risk assessment by country and tool used (n = 398).

Score	Czech Republic (n = 174)	Denmark (n = 58)	Greece (n = 70)	Netherlands (n = 57)	Sweden (n = 39)
**Heart SCORE,** *median (25–75%)*	1 (0–2)		1 (0–3)		
**Heart SCORE ≥ 5%,** *n (%)*	12 (6.9)		8 (11.4)		
**Heart SCORE ≥ 10%,** *n (%)*	4 (2.3)		3 (4.3)		
**Svenska Score,** *median (25–75%)*					0 (0–1)
**Svenska** **Score ≥ 5%,** *n (%)*					0 (0)
**Svenska Score ≥ 10%,** *n (%)*					0 (0)
**Modified Heartscore BMI score,** *median (25–75%)*		2 (1–3)			
**Modified Heartscore BMI score ≥ 5%,** *n(%)*		5 (8.6)			
**PC CMR,** *median (25–75%)*				22 (13.5–39.5)	
**PC CMR ≥ 23%** **(men) or PC CMR ≥ 19%** **(women),** *n (%)*				21 (36.8)	

Abbreviations: PC CMR = Dutch Prevention Consultation Cardiometabolic Risk.

## Supplementary Materials

The following are available online at https://www.mdpi.com/1660-4601/17/23/9080/s1. Table S1. Procedural aspects per participating country. Table S2. Demographic characteristics of participants who completed CVD-risk assessment (n = 398).
