# Supplementary material for: High Variability in Implementation of Selective-Prevention Services for Cardiometabolic Diseases in Five European Primary Care Settings
Source: Int J Environ Res Public Health. 2020 Dec 4;17(23):9080. doi: 10.3390/ijerph17239080 (PMC7730804; doi:10.3390/ijerph17239080)
Supplement: Supplementary file 1 [file ijerph-17-09080-s001.pdf]

Table 1. Procedural aspects per participating country.

	Czech Republic	Denmark	Greece	Netherlands	Sweden
Number of practices	10	2	3	5	1
Average practice size (*persons*)	1900	1600	1500	2350	20,000
Practice selection	Purposive	Purposive	Purposive	Random	Purposive
Number of invited participants	200	200	200	200	200
Participant selection	Consecutive	Random	Consecutive	Random	Consecutive
Participant age group	40–65	40–65	40–65	45–65	40–65
Participant eligibility	- No CMD diagnosis	-No CMD diagnosis<br>- No CMD treatment	- No CMD diagnosis	- No CMD diagnosis<br>- No CMD treatment	- No CMD diagnosis<br>- No CMD treatment
CVD risk assessment tool	European Heart SCORE (country adjusted): 2016 ESC/EAS Guidelines for the Management of Dyslipidemias	Modified Heartscore BMI score	European Heart SCORE (country adjusted): 2016 ESC/EAS Guidelines for the Management of Dyslipidemias	Dutch Prevention Consultation Cardiometabolic Risk (PC CMR)	Svenska Score (SCORE Sweden)
Patient approach	1-step (concurrent invitation and assessment)	2-step (separate invitation from assessment)	1-step (concurrent invitation and assessment)	2-step (separate invitation from assessment)	1-step (concurrent invitation and assessment)
Demographic characteristics	√	√	√	√	√
Physical activity	√	√	√	√	√
Smoking and alcohol consumption	√	√	√	√	√
Dietary habits	√	√	√	√	√
Participant's assessment	√		√		√

of the intervention			
Willingness to change behavior	√	√	√
Barriers to undertaking risk-reduction actions	√	√	√

Abbreviations: **CMD**: cardiometabolic disease; **CVD**: cardio-vascular disease.

Table 2. Demographic characteristics of participants who completed CVD-risk assessment (n = 398).

Variable	Czech Republic (N = 174)	Denmark (N = 58)	Greece (N = 70)	Netherlands (N = 57)	Sweden (N = 39)
**Gender**, *n (%)*					
Female	111 (63.8)	27 (46.6)	27 (37.1)	32 (55.9)	27(69.2)
Male	63 (36.2)	31 (53.4)	43 (62.9)	25 (44.1)	12 (30.8)
**Age (years)**, *mean (SD)*	49.8 (8.9)	55.7 (6.4)	53.6 (8.9)	53.2 (10.1)	51.1 (6.3)
**Education**, *n (%)*					
None	0 (0)	0 (0)	5 (7.1)	0 (0)	0 (0)
Primary	1 (0.6)	1 (2)	13 (18.6)	1 (1.8)	0 (0)
Secondary	28 (16.3)	8 (16)	35 (50)	8 (14)	0 (0)
College/University	143 (83.1)	41 (82)	17 (24.3)	48 (84.2)	39 (100)
**Work status**, *n (%)*					
Full time	117 (89.3)	35 (60.4)	38 (54.2)	29 (50.8)	36 (92.3)
Part time	29 (16.7)	10 (17.2)	13 (18.6)	16 (28.1)	2 (5.1)
Pensioner	8 (4.6)	8 (13.8)	9 (12.9)	9 (15.8)	1 (2.6)
Unemployed	4 (2.3)	3 (5.2)	10 (14.3)	2 (3.5)	0 (0)
Disabled	16 (9.2)	2 (3.4)	0 (0)	1 (1.8)	0 (0)
**Health insurance**, *n (%)*					
Yes	166 (95.4)	24 (41.4)	53 (76.9)	57 (100)	27 (69.2)
No	3 (1.7)	4 (6.9)	15 (21.7)	0 (0)	7 (17.9)
Not applicable	5 (2.9)	30 (51.7)	1 (1.4)	0 (0)	5 (12.8)
**Income compared to country's average**, *n (%)*					
Lower	33 (19.1)	20 (34.4)	46 (65.7)	4 (7)	3 (7.7)
Corresponding	55 (31.8)	21 (36.2)	11 (15.7)	32 (56)	8 (20.5)
Higher	76 (43.9)	16 (27.7)	0 (0)	19 (33.2)	24 (61.5)
Don't know	9 (5.2)	1 (1.7)	13 (18.6)	1 (1.8)	4 (10.3)

Supplementary Table S2 legend: In the Czech Republic, 10/121 (8.3%) women and 16/79 (20.3%) men did not participate in the CVD-risk assessment (p = 0.014). The mean (SD) age of participants who completed the CVD-risk assessment was 49.8 (8.9) years and of those who did not was 51.1 (7.5) (p = 0.292). In Denmark, two (6.9%) women and two (6.1%) men did not complete the CVD-risk assessment (p = 0.894). No statistically significant differences in age were observed between participants who completed versus those who did not complete the CVD-risk assessment [55.7 (6.4) vs. 55.8 (5.9) years, p = 0.950]. In Greece, 20/64 (31.3%) females and 17/43 (39.5%) males did not complete the CVD-risk assessment (p = 0.377). Mean (SD) age of participants who did not complete the CVD-risk assessment was 51.1 (7.7) years and of those who completed was 53.6 (8.9) years (p =

0.170). In the Netherlands, 3/36 (8.3%) females and 4/30 (45.5%) males did not complete the CVD-risk assessment (p = 0.511). Mean age of participants who did not complete the CVD-risk assessment was 61.1 (10.4) years and of those who completed was 53.2 (10.1) years (p = 0.378). In Sweden, everyone (100%) completed the CVD-risk assessment. Gender differences between participants completing versus those not completing the CVD-risk assessment were examined through X^2 tests, while age differences where examined through t-tests.
